# Image-guided and surgical management of breast infections: Addressing diagnostic and therapeutic challenges

**DOI:** 10.12669/pjms.42.(11AASC).15596

**Published:** 2026-04

**Authors:** Hina Pathan, Sana Zeeshan, Anam Khan

**Affiliations:** 1 Hina Pathan, MBBS, FCPS. Department of Radiology, Aga Khan University, Karachi, Pakistan; 2 Sana Zeeshan, MBBS, FCPS, FACS, MRBS. Section of Breast Surgery, Department of Surgery, Aga Khan University, Karachi, Pakistan; 3 Anam Khan, MBBS, FCPS. Department of Radiology, Aga Khan University, Karachi, Pakistan

**Keywords:** Breast infection, Breast abscess, Granulomatous mastitis, Mastitis, Tuberculous mastitis, Ultrasound-guided aspiration

## Abstract

**Background & Objectives:**

Inflammatory breast conditions encompass a broad spectrum, ranging from common acute lactational mastitis to rare chronic granulomatous diseases such as idiopathic granulomatous mastitis (IGM) and tuberculous mastitis (TBM). Chronic forms often mimic breast cancer, both clinically and radiologically, posing significant diagnostic and therapeutic challenges. The objective was to review the latest evidence on the epidemiology, pathogenesis, imaging findings, microbiology, and management of breast infections, with a focus on differentiating chronic granulomatous mastitis from malignancy and the role of minimally invasive interventions.

**Methodology:**

We searched PubMed, Embase, and Scopus for English-language studies published between January 2005 and October 2023 using keywords such as “mastitis,” “breast abscess,” “granulomatous mastitis,” “tuberculous mastitis,” “breast imaging,” and “management.” Original studies, reviews, and case series were included. Key information was extracted and summarized.

**Results:**

Lactational mastitis and breast abscesses are common and usually respond well to antibiotics or ultrasound-guided drainage. Non-lactational abscesses, IGM, and TBM are less common but often resemble malignancy. Ultrasound is the primary imaging tool, with mammography and MRI used selectively to define the extent of disease or to rule out malignancy. Minimally invasive drainage techniques are effective, providing high cure rates with superior cosmetic outcomes.

**Conclusions:**

Accurate diagnosis of breast infections requires integrated clinical, radiologic, microbiologic, and histologic assessment. Diagnosing chronic granulomatous mastitis can be challenging, especially in areas where tuberculosis is widespread. Wider use of image-guided drainage and a multidisciplinary approach can maximize results while lowering morbidity.

## INTRODUCTION

Inflammatory breast diseases comprise a wide spectrum of conditions ranging from acute conditions like lactational mastitis and breast abscesses to chronic rare entities such as idiopathic granulomatous mastitis (IGM) and tuberculosis mastitis (TBM). Both the chronic forms clinically and radiologically mimic carcinoma, causing a diagnostic and therapeutic dilemma.^1^,^2^

Accurate diagnosis requires integration of clinical evaluation with radiologic assessment, microbiologic testing, and tissue sampling, making a multidisciplinary approach essential. Over the past decade, there has been a paradigm shift away from conventional surgical incision and drainage toward minimally invasive, ultrasound-guided management approaches that reduce morbidity and preserve cosmesis.^3^,^4^

This review summarizes current evidence on epidemiology, pathogenesis, imaging features, microbiology, and management of breast infections, with particular attention to differentiating chronic granulomatous diseases from malignancy and to the relevance of minimally invasive strategies in low- and middle-income and tuberculous-endemic settings.

## METHODOLOGY

We searched PubMed, Embase, and Scopus for articles published between January 2005 and October 2023. Search terms included “mastitis,” “breast abscess,” “granulomatous mastitis,” “tuberculous mastitis,” “breast imaging,” and “management.” Original studies, systematic and narrative review articles, and case series describing epidemiology, imaging features, microbiology, and management of breast infections were included. Case reports with limited clinical relevance, non-English articles, conference abstracts, and studies lacking imaging or management data were excluded. Titles and abstracts were screened for relevance, and full texts were reviewed. After screening, approximately 25 articles were included in the final narrative synthesis. This review was based on previously published literature and did not involve direct contact with patients, access to identifiable patient data, or experimental interventions.

### Epidemiology:

Breast infections most commonly affect women aged between 18 and 50 years and remain an important cause of morbidity.^5^,^6^ In women of reproductive age, infections are predominantly lactational, typically occurring within the early postpartum period, most frequently during the first six weeks of breastfeeding. The overall incidence of mastitis can be as high as 33% in breastfeeding women, while abscesses are reported in 0.4% to 11% of breastfeeding mothers.^7^

Conversely, non-lactational abscesses are often seen in premenopausal older women and are more common in obese patients and smokers.^6^ These infections are typically subareolar and have a higher tendency to recur compared with lactational abscesses.

The chronic inflammatory conditions, Idiopathic Granulomatous Mastitis (IGM) and Tuberculous Mastitis (TM), are generally rare. Tuberculous mastitis most often affects women of reproductive age and remains uncommon, even in regions where tuberculosis is endemic.^8^ Although both IGM and TBM are uncommon, their presentation frequently overlaps with that of infectious mastitis and breast cancer, contributing to diagnostic delays and variability in reported incidence.

### Pathogenesis:

Pathogenesis varies significantly depending on the clinical context, primarily categorized into lactational (puerperal) and non-lactational forms.^5^

### Lactational mastitis:

The exact cause of mastitis in breastfeeding women remains uncertain. One widely accepted explanation is that the bacteria enter through tiny cracks in the nipple and multiply within milk-filled ducts where flow has slowed or become obstructed. Milk stasis due to ductal obstruction or nipple trauma facilitates bacterial proliferation within the lactiferous ducts. *Staphylococcus aureus* (*S. aureus*) is the organism most frequently identified in lactational mastitis. However, recent studies show that methicillin-resistant *Staphylococcus aureus* (MRSA) is becoming an increasingly common pathogen in these infections.^5^,^9^

### Non-lactational mastitis:

It can be subdivided into periductal mastitis (PDM), IGM and TBM.

Periductal mastitis (PDM) refers to inflammation centered on the subareolar ducts. The most widely accepted mechanism involves squamous metaplasia of the lactiferous ducts (SMOLD), which leads to ductal obstruction, dilation, and stagnation of secretions. Mostly sterile, this ductal stasis promotes secondary bacterial overgrowth and recurrent infection.^9^,^10^

Idiopathic granulomatous mastitis (IGM) is a rare, benign inflammatory breast disease of unknown etiology. Proposed mechanisms include autoimmune/hyperimmune responses, hormonal (hyperprolactinemia), ductal rupture with extravasated secretions, and infectious triggers. Emerging evidence implicates Corynebacterium species, particularly Corynebacterium kroppenstedtii, as a potential infectious contributor.^5^,^11^

Tuberculous mastitis (TBM) is a rare form of secondary granulomatous mastitis and usually represents a manifestation of extrapulmonary tuberculosis. Breast tissue is relatively resistant to Mycobacterium tuberculosis, which is why TBM is uncommon even in endemic regions. In most cases, breast involvement is secondary to infection elsewhere in the body. The lymphatics via retrograde spread from axillary lymph nodes is the most frequently described route. Less commonly, TBM may result from hematogenous dissemination or direct extension from adjacent structures such as the chest wall or ribs.^8^,^12^

### Imaging:

Radiology imaging reinforces the clinical examination and is particularly useful to evaluate skin thickening, the extent of inflammation, measure abscess cavities, and compare findings with the contralateral breast. It also helps guide intervention and exclude malignancy.^13^

***Ultrasound*** is the primary imaging modality in all age groups and clinical settings.^13^-^15^ Early or mild infection, such as breast cellulitis, is localized to the dermis and may be focal or diffuse.^13^,^16^ Sonographic findings may be minimal, with only subtle skin thickening.^13^,^14^ Normal skin thickness is typically ≤2 mm, and up to 4 mm in the periareolar region or inframammary folds.^13^,^15^ Using a generous amount of gel with gentle transducer pressure can improve detection of subtle changes.^13^,^14^ When dermal thickness is borderline, comparing with an unaffected quadrant or the contralateral breast can help confirm abnormality.^13^,^15^

Acute mastitis may develop from overlying cellulitis as the infection progresses deeper or may originate within the breast parenchyma without skin changes.^10^,^16^ On ultrasound, mastitis manifests as edema, with increased echogenicity of fat lobules and interspersed hypoechoic reticulations, reflecting dilated lymphatics or interstitial fluid.^13^-^15^ Increased vascularity on color doppler is usually observed reflecting hyperemia from ongoing inflammation.^13^,^14^

Persistent or untreated mastitis can progress to phlegmon or abscess. Phlegmons appear heterogeneous with surrounding hyperemia and minimal fluid, while abscesses are more liquefied, showing hypoechoic or anechoic components, sometimes with mobile debris.^14^

**Fig.1:**
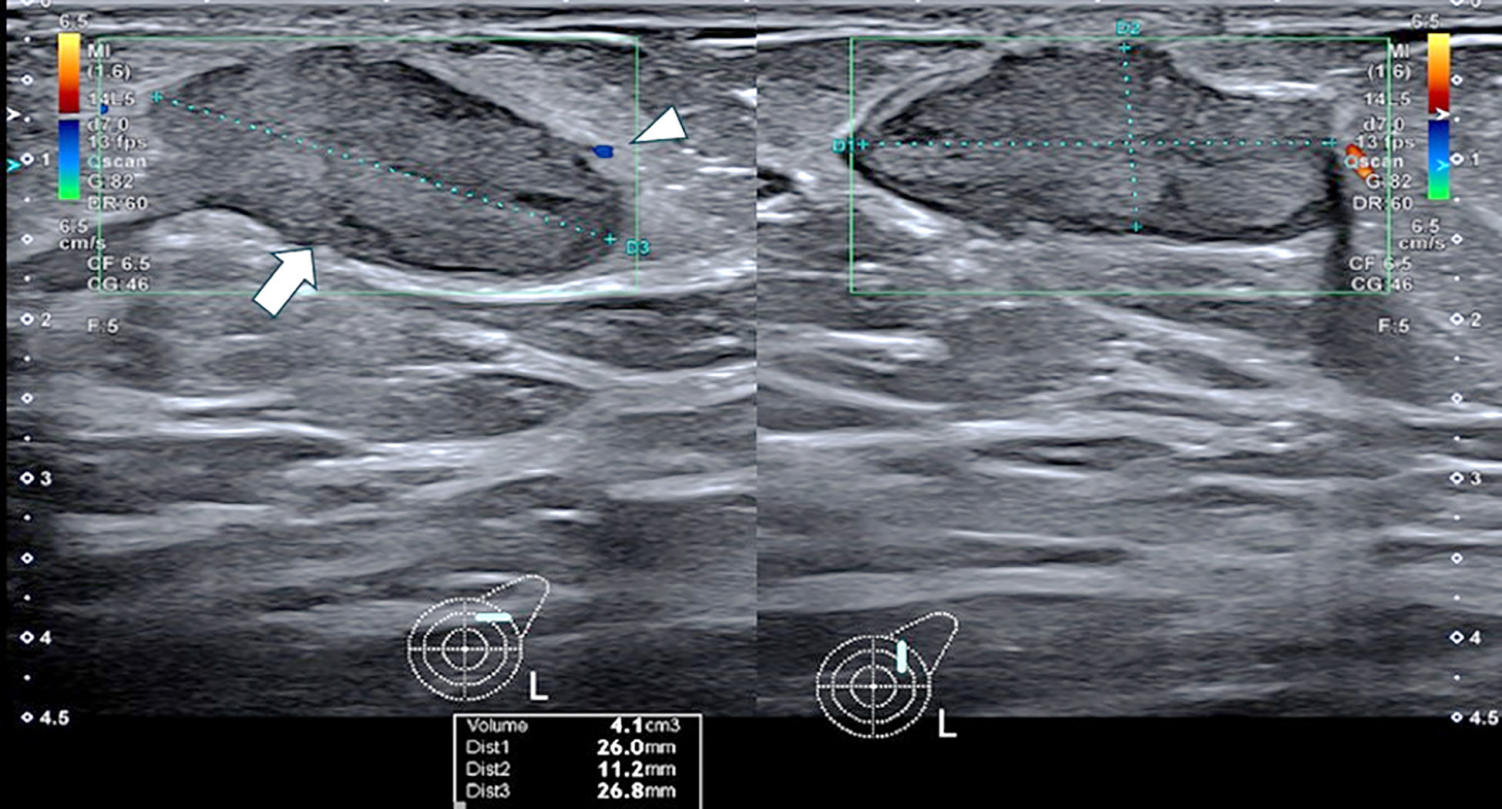
Ultrasound image of an elderly female patient with recurrent non-lactational breast abscess showing a dilated duct with homogenous, diffuse intraluminal echoes without vascularity at the 1 o’clock position (arrow). Surrounding increased echogenicity of parenchyma (arrowhead) is reactionary.

A meticulous evaluation of the lactiferous ducts for dilation, wall thickening, and intraluminal debris is essential (Fig.1). Assessment of liquefaction and loculations helps guide aspiration, and any fistulous communication with the skin should be noted.^15^

**Fig.2:**
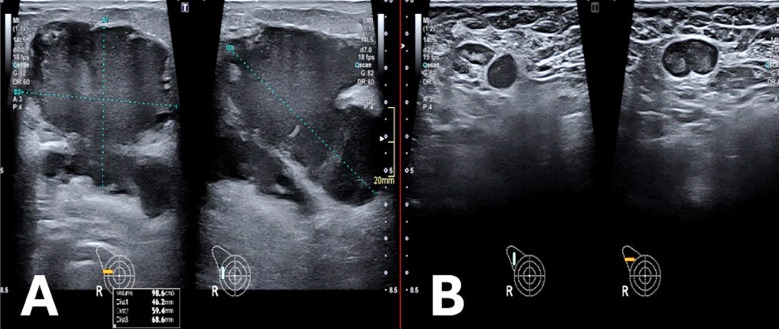
Ultrasound images of a young lactating female with breast inflammation showing. (A) fluid collection with internal echoes in right breast, consistent with lactational abscess - (B) axillary lymph node showing diffuse cortical thickening in accordance with reactive ipsilateral lymphadenopathy.

The presence of reactive axillary lymphadenopathy is common. Reactive nodes typically show preserved fatty hila and smooth, diffuse cortical thickening, whereas malignant nodes may exhibit focal or diffuse cortical thickening with loss of the fatty hilum (Fig.2).^15^,^16^

**Fig.3:**
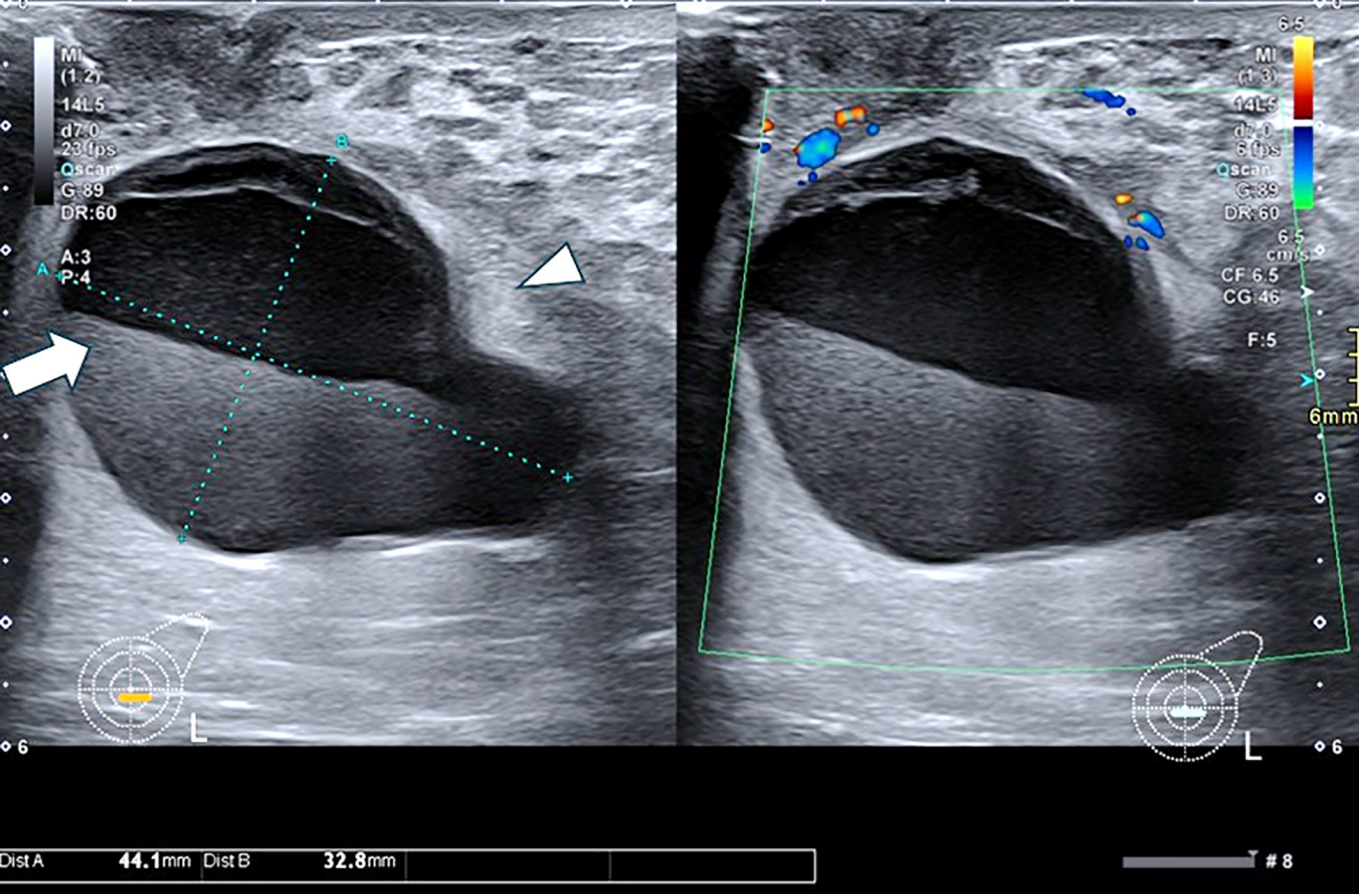
Ultrasound images of a young lactating female with a painful breast lump showing a cystic lesion with layering debris (arrow). The surrounding parenchyma shows increased echogenicity (arrowhead) and vascularity in the retroareolar region at 6 o’clock of left breast. Aspiration yielded milky fluid mixed with pus, consistent with an infected galactocele.

Infected galactocele is common in lactating patients when milk stagnates following cessation of breastfeeding, resulting in ductal dilatation and milk-filled cysts. Galactoceles may resemble abscesses but follow the ductal distribution. Diagnosis is usually confirmed by aspiration of milky fluid which may be mixed with pus (Fig.3).^6^

**Fig.4:**
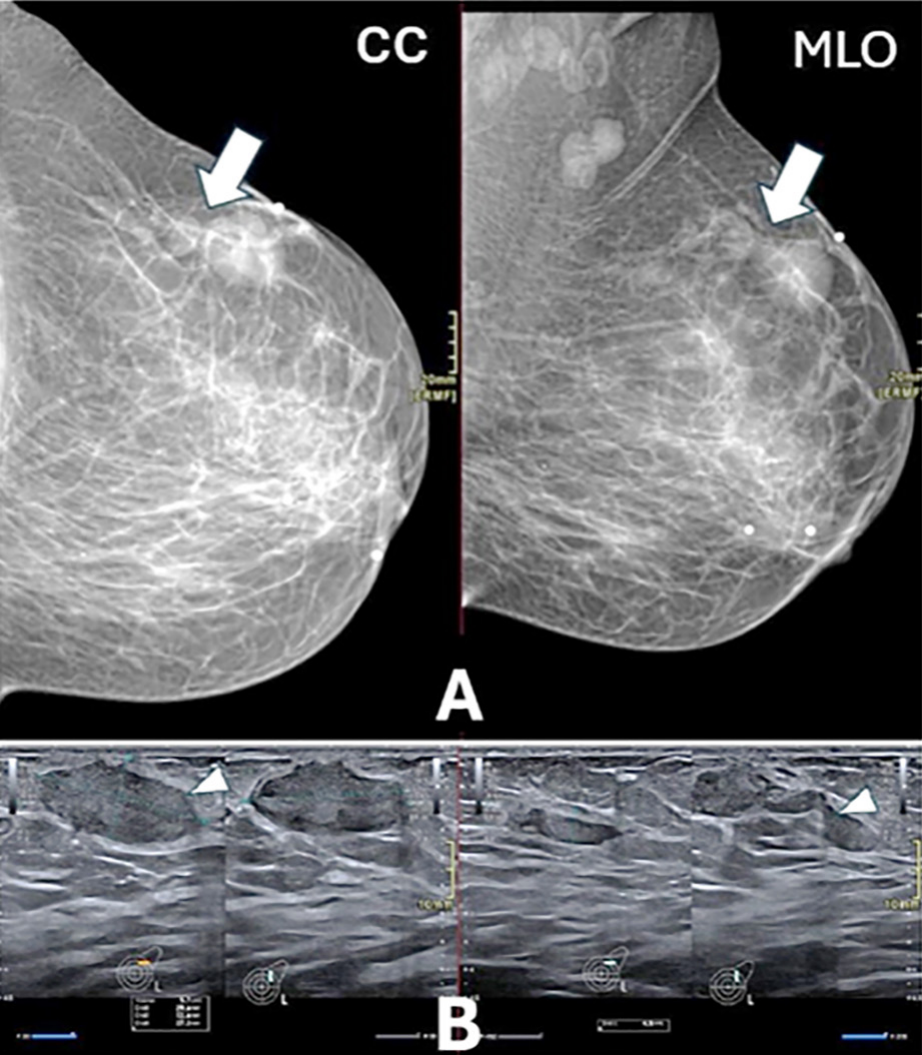
Radiographic images of an elderly female with a painful breast lump. (A) Left breast mammogram (CC and MLO views) showing two partly defined high density nodules (arrows) in the upper outer quadrant of left breast underlying the site of metallic marker and (B) Ultrasound of the same patient shows intercommunicating pockets of collection with internal homogenous avascular echoes.

***Mammography*** is mainly useful in non-lactating patients or when malignancy is suspected. (Fig.4) Mammographic findings are not specific and could be focal or diffuse. It can show skin thickening, focal asymmetry or mass, architectural distortion, nipple retraction, and axillary lymphadenopathy.^15^ The absence of mammographic abnormalities does not obviate the need for targeted ultrasound, as mild mastitis may be inapparent on standard views and image quality may be limited in patients unable to tolerate compression. Use of mammography is limited in young and lactating women because of dense parenchyma.^17^

***Magnetic Resonance Imaging (MRI)*** *is* not routine but is highly sensitive in problem-solving. It is invaluable for diagnosing complicated fistulous tracts, evaluating chest wall invasion, and distinguishing benign mastitis from inflammatory breast cancer.^13^ Dynamic contrast-enhanced MRI can help by demonstrating a slower, gradual enhancement pattern in mastitis compared to the rapid wash-in and wash-out kinetics often seen in malignancies. MRI offers an advantage over ultrasound in detecting deeper abscesses, especially in large or dense breasts. It is also valuable for evaluating subareolar abscesses that may cause nipple inversion, including smaller lesions that are difficult to visualize on ultrasound.^14^

### Management:

Management is tailored to the nature and severity of the infection.

### Lactational Mastitis and Abscesses:

For uncomplicated mastitis, prompt antibiotic therapy (targeting *S. aureus*) and continued breastfeeding or milk expression are essential. For abscesses >3 cm, ultrasound-guided aspiration is now the first-line intervention. Multiple aspirations may be required. This approach yields success rates exceeding 85-90% with excellent cosmetic outcomes, making it superior to traditional incision and drainage (I&D) for most cases.^7^,^9^,^18^ Antibiotic therapy is guided by culture results, breastfeeding status, and severity of infection. Courses generally last five to seven days but may be extended in recurrent or severe cases. Hospitalization is indicated for large abscesses or patients showing systemic signs of infection, with intravenous antibiotics and I&D as needed. Pain management typically involves nonsteroidal anti-inflammatory and, if necessary, short-term narcotics.^18^,^19^

Local studies from Pakistan support this shift: Manzoor A et al. reported shorter healing times, higher continued breastfeeding rates, and better cosmetic results with needle aspiration compared to I&D.^20^ A study conducted by Sushel C et al. reported that ultrasound guided aspiration with oral antibiotics is effective, safe and practical for early treatment for lactational breast abscess, together with encouraging patients to continue breast feeding.^21^

### Non-Lactational Abscesses:

Underlying conditions like PDM, IGM or TBM should be considered in cases of non-lactation or recurrence. These patients are often present with a firm, unilateral mass, sometimes accompanied by abscess or fistula formation. Biopsy for histopathology and gene Xpert are required in cases of recurrence or refraction to standard treatment.

PDM mostly resolves spontaneously or may require non-steroidal anti-inflammatory drugs.^5^ For IGM, management remains controversial and is primarily medical. **Corticosteroids** are the cornerstone of treatment for moderate to severe IGM, leading to resolution in most cases.^3^ Methotrexate or other immunosuppressants can be used for steroid-resistant cases. Antibiotics may be used if secondary infection is suspected or if *Corynebacterium* is identified. Surgical excision is reserved for limited, localized disease or medically refractory cases but carries a risk of poor wound healing and sinus tract formation.^4^

Latif H et al. emphasized the diagnostic dilemma associated with IGM in the local population, noting that misdiagnosis and delayed recognition are common, particularly because clinical and imaging features can mimic malignancy or infectious mastitis. Their study highlighted that recurrence is frequent despite corticosteroid therapy, and careful follow-up is essential. They also reported that a combination of medical therapy and limited surgical intervention, guided by histopathology, achieved favorable outcomes while minimizing complications.^22^

***Treatment for TBM*** involves standard anti-tuberculous therapy (ATT) for 6-12 months. Image-guided aspiration or biopsy is crucial for obtaining tissue for culture and histology. Surgical intervention is typically limited to drainage of large abscesses or diagnostic excision when needed.^8^ Local studies support the effectiveness of this approach. Afridi highlighted that early recognition and medical management with ATT were sufficient for most patients, with surgical intervention needed in a minority of cases, usually for diagnostic purposes or management of large abscesses.^23^ These findings underscore that, in TBM, prompt diagnosis and adherence to medical therapy are key to favourable outcomes while minimizing unnecessary surgery.

## CONCLUSION

Accurate diagnosis of breast infections requires a comprehensive, multidisciplinary approach combining clinical, radiologic, microbiologic, and histopathologic findings. Chronic granulomatous conditions pose significant diagnostic challenges due to their overlap with malignancy. Ultrasound is the cornerstone of imaging, while mammography and MRI serve as valuable adjuncts in selected cases. The management paradigm has decisively shifted towards minimally invasive, image-guided percutaneous techniques for abscess drainage, which should be considered the standard of care due to their high efficacy and superior cosmetic results. In TB-endemic and resource-constrained settings, clinicians should adopt a structured diagnostic pathway that includes early image-guided core biopsy with mycobacterial studies in cases of atypical or non-resolving breast inflammation, and prioritize percutaneous drainage combined with targeted antimicrobial therapy as a first-line intervention. Increased awareness and early implementation of these strategies are essential to reducing diagnostic delays, minimizing morbidity, and improving overall patient outcomes.

### Authors’ Contributions:

**HP:** Conception of the study, literature review, and drafting of the manuscript.

**SZ** Critical revision of the manuscript for important intellectual content and literature review.

**AK:** Conception of the study, literature review, drafting of the manuscript and responsible for coordinating responses.

All authors have approved the final version of the manuscript.
